# Impact of Synergistic Association of ZnO-Nanorods and Symbiotic Fungus *Piriformospora indica* DSM 11827 on *Brassica oleracea* var. botrytis (Broccoli)

**DOI:** 10.3389/fmicb.2017.01909

**Published:** 2017-10-17

**Authors:** Uma Singhal, Manika Khanuja, Ram Prasad, Ajit Varma

**Affiliations:** ^1^Amity Institute of Microbial Technology, Amity University, Noida, India; ^2^Centre for Nanoscience and Nanotechnology, Jamia Millia Islamia, New Delhi, India

**Keywords:** zinc oxide nanorods, *Piriformospora indica*, UV-vis, XRD, SEM, confocal microscopy

## Abstract

In the present work, novel nanotool called ‘nano-embedded fungus’ formed by impact of synergistic association of ZnO-nanorods and fungus *Piriformospora indica* DSM 11827, for growth of *Brassica oleracea* var. botrytis (Broccoli) is reported. ZnO-nanorods were synthesized by mechanical assisted thermal decomposition process and characterized by scanning electron microscopy (SEM) for morphology, X-ray diffraction for structural studies and UV-vis absorption spectroscopy for band gap determination. Nanoembedded fungus is prepared by optimizing ZnO-nanorods concentration (500 ppm) which resulted in the increased biomass of *P. indica*, as confirmed by dry weight method, spore count, spread plate and microscopy techniques viz. SEM and confocal microscopy. Enhancement in *B. oleracea* var. botrytis is reported on treatment with nanoembedded fungus. According to the authors, this is the first holistic study focusing on the impact of ZnO-nanorods in the enhancement of fungal symbiont for enhanced biomass productivity of *B. oleracea* plant.

## Introduction

Agricultural nanotechnology has the potential to overcome the challenges associated with undeveloped farming practices including unbalanced ecosystem and low productivity through nano-formulation of fertilizers (or pesticides, herbicides), effective management of soil and water resources through porous nanostructures. This leads to enrichment in nutritional quantity as well as quality, simultaneously rejuvenating soil fertility and stabilization of erosion-prone surfaces (Tebebu et al., 2015; Prasad et al., 2017).

Zinc oxide (ZnO) is considered to be one of the best exploited materials at nano dimensions because of its large excitonic binding energy and wide band gap which is important for both scientific and industrial applications (Wang et al., 2004; Bhuyan et al., 2015a; Kotzybik et al., 2016; Baral et al., 2017). ZnO nanostructures exhibit gigantic area of applications and potential to boost the yield, development of food crops and their use as food additive (Sawai et al., 1996; Huang et al., 2001; Song et al., 2006; Bhuyan et al., 2015a,b; Wang et al., 2015; Sharma et al., 2017). ZnO is currently listed as “generally recognized as safe (GRAS)” material by the Food and Drug Administration (Rajiv et al., 2013). In previous reports, the colloidal solution of zinc oxide is used as ‘nano-fertilizer’ a plant nutrient which is more than a fertilizer because it not only supplies nutrients for the plant but also revives the soil to an organic state without the harmful factors of chemical fertilizer (Sabir et al., 2014; Taheri et al., 2016). *Piriformospora indica* DSM 11827 is a multifunctional fungus, recently named as *‘Serendipita indica’* acts as plant growth promoter, biofertilizer, metabolic regulator, bio-herbicide, immunomodulator, phytoremediator, bio-insecticide and bio-pesticide, antioxidant enhancer, etc. (Prasad et al., 2013; Gill et al., 2016). It has proven attributes for enhanced plant productivity and confers resistance against biotic (Andrade-Linares et al., 2013; Ansari et al., 2013) and abiotic stresses (Franken, 2012; Gill et al., 2016; Weiß et al., 2016).

This study targets to develop a nanotechnology-assisted fungal symbiont with an objective to enhance crop productivity and medicinal value of human food crops viz. *Brassica oleracea* to overcome the challenges associated with the conventional farming (Singh et al., 2016). In the present study ZnO-nanorods have been synthesized, and the impact on fungal symbiont was studied by optimizing ZnO-nanorod concentration on interaction with *P. indica* which results in enhanced biomass. The optimized nanorods interacted fungal symbiont is called “Nanoembedded fungus.” Study was further performed on Broccoli (*Brassica olearacea* var. botrytis) plants. In this study, effect of ZnO-nanorod embedded *P. indica* was analyzed on *B. oleracea* plants. Two treatments were given to *B. oleracea* plants in triplets that are *B. oleracea* treated with (i) *P. indica*, (ii) ZnO-nanorod embedded *P. indica* and the plants without any treatment were taken as control.

## Materials and Methods

### Experimental

Chemicals of analytical grade were used in all the experiments, directly without any further purification, procured from Sigma–Aldrich (India), Merck (India), and HiMedia (India). In all the conducted experiments, Millli-Q water or double distilled water (ddH_2_O) was used. Glassware was rinsed with Milli-Q water and air-dried before use in experiments.

Pure ZnO-nanorods were prepared by mechanical-assisted thermal decomposition process (Bhuyan et al., 2015a,b). In ZnO-nanorods synthesis process, 2 gm of zinc acetate dihydrate [Zn (CH_3_COO)_2_.2H_2_O] was grinded in mortar pestle for 45 min. The grinded powder was placed in an alumina crucible and heated in programmable furnace (ramp rate 4°C/min) at 300°C for 4 h. Therefore, the synthesis process is termed as mechanical assisted thermal decomposition process. Double distilled water was used to wash the resultant powder twice followed by drying in hot air oven at 100°C for 8 h.

### Characterization

Zinc oxide-nanorod structure and surface morphology of the samples were observed using scanning electron microscopy (SEM) (Model: JEOL-JSM-6010LA) at an accelerating voltage of 20 kV. The absorption spectrum was measured by Perkin-Elmer Lambda 35 UV-vis spectrometer. Band gap of the sample were calculated using Tauc’s plot. X-ray diffractometer (Model: Bruker; D2-Phaser) was used to investigate crystalline structures of ZnO-nanorod. The diffractogram was recorded in the scan range of 5 to 80° using CuK_α_ (λ = 1.5403 Å) X-ray operated at10 kV, 30 mA.

### Fungal Strain and Culture Conditions

Aspergillus medium (Hill and Kaefer, 2001; Prasad et al., 2013) was found to be the best among different synthetic media to grow the axenically grown fungus *P. indica* DSM 11827. Circular solidified disks (4 mm dia.) consisting of actively grown hypha and chlamydospores of *P. indica* were placed on solidified aspergillus medium (pH 6.8–7.0, 28 ± 2°C in dark) as well as in broth. After 7 days, the Petri plates were found to be completely filled up with the fungal biomass.

#### Scanning Electron Microscopy (SEM) and Energy Dispersive X-ray Spectroscopy (EDX)

Scanning electron microscopy (EVO 18 Special edition, ZEISS) analyses were carried on *P. indica* before and after treatment with ZnO-nanorods. The *P. indica* culture without ZnO-nanorods treatment was taken as control. The chemical fixation of *P. indica* was done in order to stabilize and preserve its chemical structure. *P. indica* disks were washed with 0.1 M sodium phosphate (pH 7.4) buffer for 30 min at room temperature, then put the sample in fixative, i.e., 2.5% glutaraldehyde for overnight. In order to remove the glutaraldehyde deposits, the suspension was sequentially washed with 0.1 M sodium phosphate buffer solution (pH-7.4) and distilled water, followed by centrifugation for further isolation. Sample was dehydrated with ascending series from 50 to 100% ethanol (EtOH), in 10% increments for 20 min each and finally kept for drying. The elemental analysis of the sample was carried with energy dispersive X-ray (EDX) spectroscopy facility (Oxford instruments, 51-ADD0048) using SMARTSEM software to confirm the presence to zinc oxide nanorods in the treated sample.

#### Dry Weight Method

The growth of fungal biomass on interaction with ZnO-nanorods, was signified in terms of increase in dry weight. *P. indica* was inoculated in 100 ml of Hill & Kaefer medium. The culture was incubated in dark (28 ± 2°C, 80 rpm). ZnO-nanorods in different concentrations (ppm) viz. 300, 400, 500, 600, 1000, and 2000 were added to the culture after 3 days of incubation. The culture was incubated for 4 more days. *P. indica* without any treatment was grown separately for maintaining control against treated sample. Fungal culture was filtered after 7 days of incubation. The dry cell weight was calculated using:

$$W=\left[\frac{C-C_{0}}{C_{0}}\right]\times 100$$

where, W is increase in dry weight of fungal biomass on treatment with ZnO-nanorods, C_o_ is dry weight of fungal biomass without any treatment called ‘control’ and *C* is the dry weight of ZnO-nanorods treated fungal biomass. The concentration of ZnO-nanorods for which maximum fungal biomass was obtained is termed as ‘optimized ZnO.’

#### Quantification of Spore Using Hemocytometer

Spores were harvested from *P. indica* cultured on agar plate by flooding the culture with 5 ml of 0.05% (v/v) ‘Tween 80’ solution. The spores were carefully scraped off from the hyphae using sterile glass spreader. Spores were collected in 15 ml centrifuge tube and centrifuged for 5 min at 800 rpm to remove left over hypha fragments. Supernatant was discarded and pelleted spores were counted using hemocytometer.

#### Quantifying Colonies and to Study Their Morphology by Spread Plate Technique

*Piriformospora indica* culture was suspended in the test tubes containing distilled water with the dilution factor of (10^-1^, 10^-2^, 10^-3^ upto 10^-7^ respectively). 1 μL of suspension (10^-7^) and 1 mL of optimized ZnO-nanorods was poured onto the agar plates (triplicates). The prepared suspension was spread and incubated for 7 days at 28 ± 2°C. Petri plates without nanorods were taken as control.

#### Confocal Microscopy

*Piriformospora indica* culture with and without ZnO-nanorods were observed under a confocal laser scanning microscope LSM-780 (Carl-Zeiss, Inc., Jena, Germany). For culture staining, wheat germ agglutinin Alexa Fluor- 488 (WGA-AF488, Molecular Probes, Eugene, OR, United States) was used. Ethanol/chloroform/trichloroacetic acid in the ratio 1/4/0.15% v/v/w were used for the fixation of fungal biomass. The culture was washed three times with distilled H_2_O, boiled in 10% KOH for 1 min, washed with phosphate buffered saline (PBS). Afterward, biomass was stained with PBS solution containing 0.2% Silwet L-77 and 50 μg/mL WGA- AF488. Vacuum infiltration of treated and control biomass in staining solution was done three times under 50 mm Hg vacuum. The cultures were transferred to PBS followed by removal of staining solution. The cultures were analyzed under confocal microscope, fungal hyphae was strained with WGA-AFA 488.

Additionally, trypan blue was used to stain *P. indica* spores and hyphae, on binding to cells strong red fluorescence was radiated. Trypan blue staining facilitated quantification of cell size and cell wall volume under confocal microscope, thus enabling the quantification of morphological changes viz. spore size, hyphae thickness.

#### Treatment of *B. oleracea* with Nanoembedded Fungus

*Brassica oleracea* seeds brought from Indian Agriculture Research Institute (IARI), New Delhi, India of same sizes were planted in pots. The average germination rate of the seeds was 75% as shown on MS medium (Murashige and Skoog, 1962). To minimize errors in seed germination and seedling vigor, the seeds of uniform size were selected. The plants were incubated under humidity (60%), temperature (24 ± 2°C) and light (1000 lux, 16 h light and 8 h dark). After 15 days of exposure, roots and shoots were separated and washed with water to remove the growth medium and dried with wipes to remove the surface water. The growth parameters like stem and root length, fresh and dry weight per plant were recorded.

### Statistical Analysis

Each treatment was conducted in triplicates and the results were presented as mean ± standard error (SE) and analyzed by using one-way ANOVA.

## Results

**FIGURE 1:**
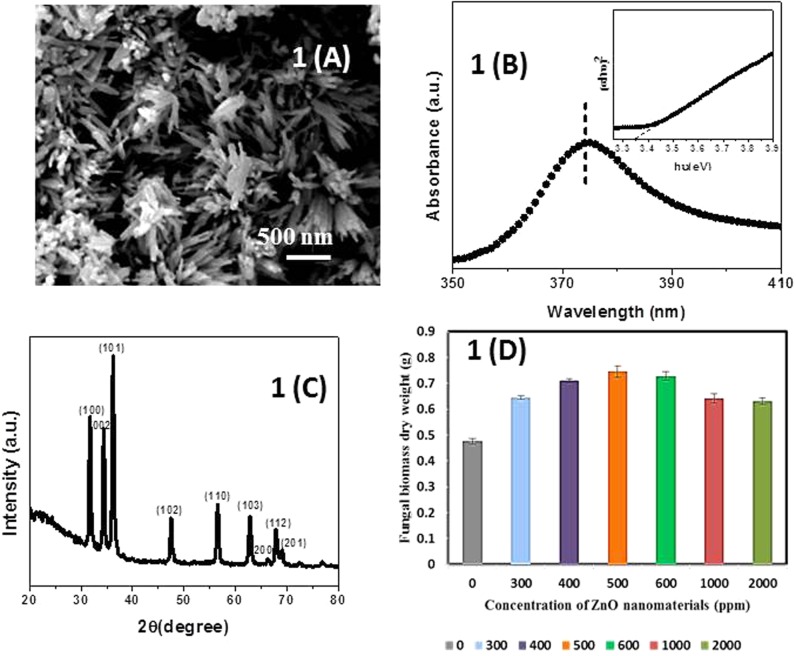
**(A)** Scanning electron microscopy (SEM) micrograph of pure ZnO-nanorods. **(B)** Tauc’s plot and UV-vis absorption spectra (inset of **B**) of pure ZnO-nanorods. **(C)** X-ray diffraction (XRD) pattern of pure ZnO-nanorods. **(D)** Histogram showing dry weight of *Piriformospora indica* after incubation with different concentrations of ZnO-nanorods. Data represented as mean ± standard error.

**Figure 1A** showed the SEM of ZnO-nanorods. The ZnO-nanorods were of an average diameter 50 nm and length of 500 nm. **Figure 1B** showed the UV-vis absorption spectra of pure ZnO-nanorods. Band gap is calculated by Tauc’s relation: a = A(hυ -E_g_)^n^/hυ, where a is the absorption coefficient, A is constant, E_g_ is the absorption band gap, n is subjected to the nature of the transitions, n may have values 1/2, 2, 3/2, and 3 corresponding to allow (direct and indirect), forbidden (direct and indirect) transitions, respectively. In this case, *n* = 1/2 for direct allowed transition. The band gap of 3.35 eV was obtained from the Tauc’s plot (inset of **Figure 1B**).

**Figure 1C** shows the X-ray diffraction (XRD) pattern of pure ZnO-nanorods. After comparing with JCPDS File number (06-82151), all the peaks were labeled with (hkl) planes. Wurtzite structure for ZnO-nanorods was confirmed by XRD pattern. The lattice constants ‘a’ and ‘c’ can be calculated using the relations (a) and (b) given below:

$$a=\sqrt{\frac{1}{3}}\frac{\lambda }{\sin\;\theta }\,\;\;\;\;\;\;\;\;\;\;\;\;\;\;\;\;\;\;\;\;\left(a\right)$$

$$c\,\;=\;\sqrt{\frac{\lambda }{\sin\,\;\theta }}\;\;\;\;\;\;\;\;\;\;\;\;\;\;\;\;\;\;\;\;\;\;\;\;\;\;\;\;\;\;\;\;\left(b\right)$$

where, λ is the wavelength of incident X-ray beam and theta (𝜃) is angle of incidence.

The lattice constant ‘a’ and ‘c’ of the wurtzite structure of ZnO-nanorod were found to be 3.247 and 5.203 Å, respectively.

### Dry Weight

Fresh culture of *P. indica* was treated with ZnO-nanorods of various concentrations of 300, 400, 500, 600, 1000, and 2000 ppm, respectively. The untreated culture was taken as control. The study was conducted in triplicates. The cultures were incubated at 27°C for 8 days. After that the samples were filtered by using Whatman filter paper, and followed by drying of filtered biomass at 70°C in hot air oven for 24 h. The dry weight of the samples was taken by using the metal balances (Balance AE240 Metler). The fungal biomass observed to be 35.5, 49.2, 56.5, 52.9, 34.7, and 32.6% for ZnO-nanorod concentrations of 300, 400, 500, 600, 1000, and 2000 ppm, respectively. The best growth, i.e., 56.4% was observed when the fungus was incubated with 500 ppm of ZnO-nanorods concentration (**Figure 1D** and **Table 1**). The 500 ppm concentration of ZnO-nanorods is regarded as ‘optimized ZnO-nanorods’ for interaction with *P. indica*. In all followed studies, *P. indica* is treated with optimized ZnO-nanorods.

**Table 1:** Dry weight of *Piriformospora indica* after addition of different concentrations of ZnO-nanorods.

ZnO-nanorods {concentration (ppm)}	Fungal biomass {dry weight (gm)}	Increase in biomass {dry weight (%)}
0	0.48	
300	0.65 ± 0.0065	35.5
400	0.71 ± 0.0062	49.16
500	0.75 ± 0.0226	56.51
600	0.73 ± 0.0156	52.94
1000	0.64 ± 0.0180	34.66
2000	0.63 ± 0.0121	32.56


*Each value is mean ± standard error of three replicates*.

### Morphological Changes in Fungal Spores When Treated with ZnO-Nanorods

**FIGURE 2:**
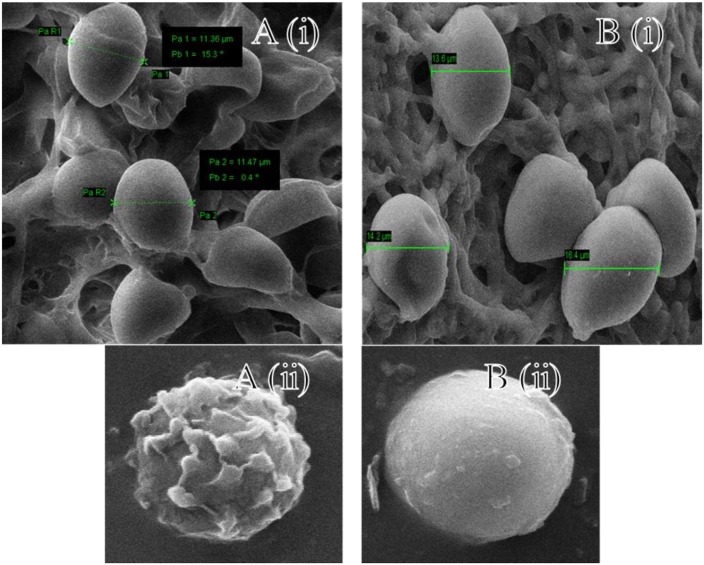
Scanning electron microscopy images: **A(i)** control and **B(i)** ZnO-nanorods treated *P. indica*; **A(ii)** and **B(ii)** shows the magnified view of spores in control and ZnO-nanorods treated *P. indica* respectively.

The changes in the size and spores count of the primed fungal samples were analyzed by SEM as shown in **Figure 2**. The SEM images of the fungal culture clearly show pear-shaped chlamydospores. In control, spores were small (diameter ∼ 11.4 μm) and less in number whereas in ZnO-nanorods treated *Piriformospora indica* spores were large (diameter ∼ 16.4 μm) and more in number as shown in **Figures 2A(i),B(i)**, respectively. **Figures 2A(ii),B(ii)** showed the magnified view of spores in control and ZnO-nanorods treated *P. indica*, respectively. As evident, in control sample spores were rough whereas in ZnO-nanorods treated *P. indica*, spores were smooth.

**FIGURE 3:**
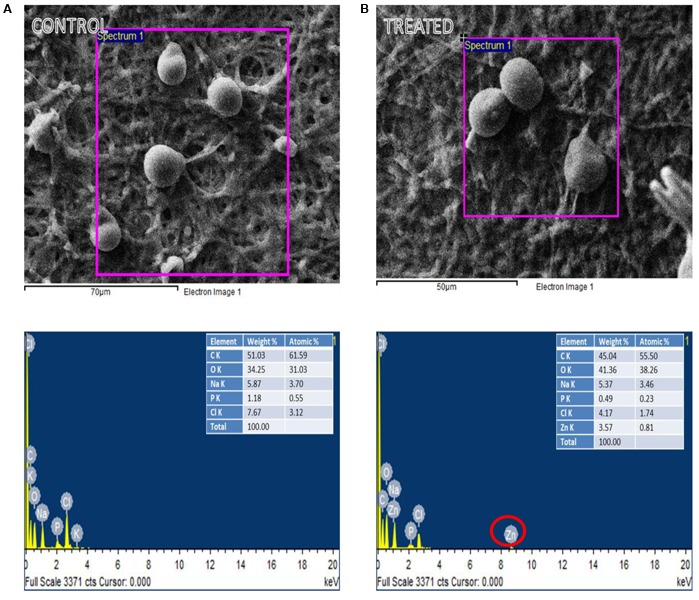
EDX spectra of the selected region of: **(A)** control and **(B)** ZnO-nanorods treated *P. indica* (red circle highlights the presence of Zinc in treated sample).

**Figures 3A,B** shows the EDX of control and ZnO-nanorods treated *P. indica* samples, respectively. Elemental analysis confirmed the presence of Zinc in ZnO-nanorods treated *P. indica* sample as indicated by peak at 8.6 keV. Inset in the figures shows the atomic and weight percent of all the elements like C, Cl, P, Na, and O present in both the samples and Zn in the treated sample.

### Spore Count Using Hemocytometer

**FIGURE 4:**
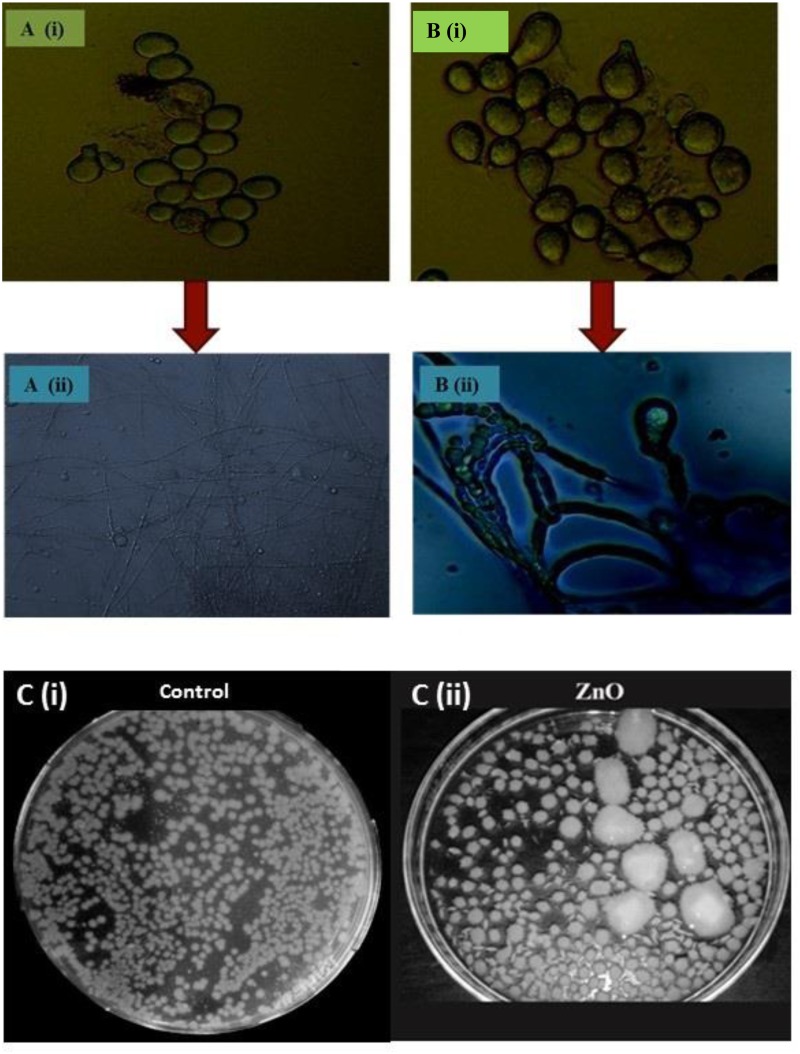
Comparative analysis of *P. indica* spores using light microscopy: **(A)** Without staining **(i)** Control and **(ii)** ZnO-nanorods treated *P. indica*, showing more number of bigger spores. **(B)** With staining using trypan blue **(i)** control and **(ii)** ZnO-nanorods treated *P. indica*, shows the morphogenesis of hyphae and spores with early sporulation as compared to control. **(C)(i)** *P. indica* ‘control’ and **(ii)** ZnO-nanorods treated *P. indica*.

Spore counts studies were carried out using hemocytometer. In control, the fungus yield was 5.34 ( ± 0.28) × 10^9^ spores/ml and in ZnO-nanorod treated *P. indica* sample; significant increase in sporulation viz. 7.18 ( ± 0.32) × 10^9^ spores per ml (*P* < 0.0001) were observed. **Figures 4A(i,ii),B(i,ii)** showed control (*P. indica* grown without any treatment) and ZnO-nanorod treated *P. indica*, respectively under light microscope. In control, spores were small in size and less in number, and hyphae is thin walled as shown in **Figure 4A(i,ii)**. On the other hand, the large and more number of bigger spores and early sporulation were observed in ZnO-nanorods treated *P. indica* as shown in **Figure 4B(i,ii)**. Based on the dry weight results, ZnO-nanorods (500 ppm) termed as ‘optimized ZnO’ is selected as a stimulatory agent for growth enhancement of *P. indica* and to study the effect of ZnO-nanorods treated *P. indica* on the growth of *B. oleracea*.

### Spread Plate Technique

Effect of optimized ZnO-nanorods on *P. indica* is also studied by spread plate technique. **Figure 4C(i)(ii)** shows 7 days cultured *P. indica* (control) and ZnO-nanorods treated *P. indica* in Petri plates, respectively. In control, small with only 235 ( ± 0.26) distinct colonies were observed whereas large number viz. 270 ( ± 0.21) of bigger distinct colonies with an overall 50% enhancement of fungal biomass in ZnO-nanorods treated *P. indica* were observed.

### Confocal Microscopy

**FIGURE 5:**
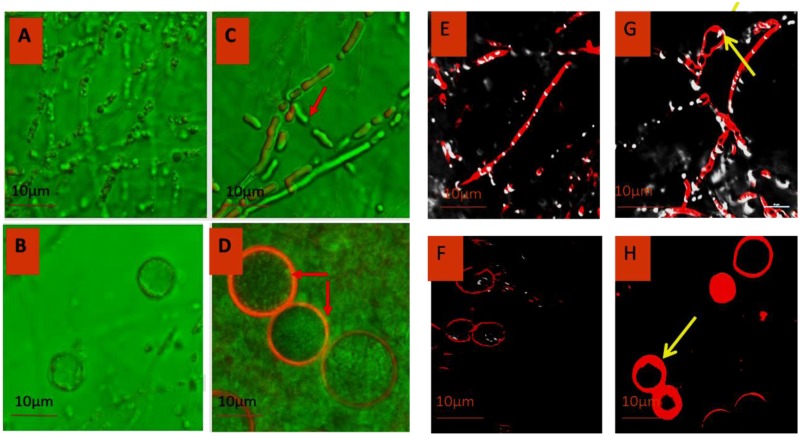
Comparative morphology analysis of *P. indica* before **(A,B)** and after treating it with ZnO-nanorods **(C,D)** by Confocal microscopy using Alexafluor 488 dye and similar analysis was done by using Trypan Blue dye before **(E,F)** and after treatment **(G,H)** with ZnO-nanorods [red arrow shown the thick hyphae as compared to control and early sporulation **(G)**].

Morphology of *P. indica* was viewed under confocal laser scanning microscopy (CLSM) using Alexa fluor 488 (**Figures 5A–D**) and trypan blue (**Figures 5E–H**). Laser excitation at 488 nm resulted in emission in the visible range. In the control specimen, the hyphae (**Figures 5A,E**) were thin walled; spore (**Figures 5B,F**) count was low with morphological deformities and disaggregation. In ZnO-nanorods treated *P. indica*, the hyphal walls were thick and hyaline (**Figures 5C,G**); spores (**Figures 5D,H**) were large in size, count was more with smooth surface topology.

### Interaction with *Brassica oleracea*, *P. indica* and ZnO-Nanorods

**FIGURE 6:**
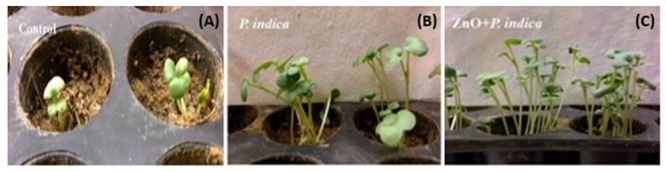
*Brassica oleracea* plants treated with fungus and nano embedded fungus: **(A)** Control, i.e., plants without any treatment, **(B)** *P. indica*, and **(C)** ZnO-nanorods + *P. indica*.

Two treatments were given to *B. oleracea* plants in triplets, *P. indica*, and ZnO-nanorods treated *P. indica* called ‘nanoembedded fungus’ and the plants without any treatment were taken as control. *B. oleracea* responded variably toward the treatments, results are summarized in **Table 2**. Seeds treated with nanoembedded fungus recorded significant germination rate, significant increase in dry and fresh weight as well as prominent increase in root and shoot length as compared to other treatments as mentioned in **Table 2** and **Figure 6**.

**Table 2:** Summarization of effect on *Brassica oleracea* plants when treated with (i) *P. indica* and ZnO-nanorods + *P. indica* in terms of various parameters.

Parameters	Control	*P. indica*	*P. indica* + ZnO-nanords
Seed germination rate ( ± SE)	18.66 ± 1.15^a^	20.33 ± 1.52^b^	23 ± 1^c^
Shoot length (cm) ( ± SE)	5.23 ± 0.02^a^	6.87 ± 0.02^b^	10.46 ± 0.1528^c^
Root length (cm) ( ± SE)	1.51 ± 0.015^a^	1.68 ± 0.02^a^	2.52 ± 0.02^b^
Fresh weight (g) ( ± SE)	5.5 ± 0.2^a^	6.13 ± 0.3^b^	11.73 ± 0.32^c^
Dry weight (g) ( ± SE)	0.933 ± 0.15^a^	1.2 ± 0.1^b^	3.36 ± 0.25^c^


*The results of treatments were compared with control. Data are mean ± standard error of three replicates. Values with different letters within same column show significant differences at P < 0.05 level between treatments according to the Duncan’s multiple range test*.

## Discussion

The dry weight results indicated that the fungal biomass was heightened approximately two times on interaction with optimized ZnO-nanorods (500 ppm) as compared to the control. The spores were more in number, large sized, smooth and round in ZnO-nanorod treated *P. indica* whereas in control sample, spores were less in number, small sized and rough as evident from SEM and confocal microscopy studies. SEM clearly indicated the stimulating effect on the size of the chlamydospores. The increase in size of the spores was almost 50% in comparison to the control. The results also exhibited the increased spore density of the test fungus on interaction with ZnO-nanorods.

Availability of different nutrients causing diauxic growth attributes to the observed results, as alternative pathways are activated beyond a certain physiological threshold value. The infused nanomaterial of different concentrations leads to the varied stress compensation pathway stimulation (Kotzybik et al., 2016). The interaction between the nanoparticle surface and cell wall impacts tremendously on dispersion of the nutrients and hence affects the growth rate (Suman et al., 2010; Ren et al., 2011; Feng et al., 2013). Researchers reported that cells when subjected to oxidative stress, leads to stimulation of stress compensation mechanisms, making them competent at sub-optimal growth conditions, as compared to those cells which were not pre-stressed (Fillinger et al., 2001; Alvarez-Peral et al., 2002; González-Párraga et al., 2003; Tripathi et al., 2016a,b, 2017).

It was observed that the stage of nanomaterials inclusion is essential. The antimicrobial property of nanomaterials was recorded when added at a late growth phase. However, the inclusion before media sterilization showed a positive and stimulating effect on the fungus, as the case with our present studies. Researchers (Aguilar-Uscanga and Francois, 2003; Siddhanta et al., 2016) reported thick walled hyphae and large number of spores of bigger size on interaction of nanomaterials with *P. indica* as evident by SEM and CLSM studies (Schermelleh et al., 2010). Roncero and Durán (1985) had observed that ZnO-nanorods treated cells were resulted in added multi-cellular aggregates with early germination of spores and abnormally thick septa.

The conflicting effect of nanomaterials at a specific stage of addition can be best explained by considering the mechanism of antimicrobial action. Antimicrobial behavior of the nanoparticles is reported to be due to the presence of electronic effects brought about as a result of changes in the local electronic structure of the surfaces due to small sizes (<100 nm) (Sharma et al., 2016). Nanomaterials, especially silver nanoparticles, strongly interact with the thiol groups of the vital enzymes and inactivate them (Aziz et al., 2015, 2016; Patra and Baek, 2017; Prasad et al., 2017). As a result, the DNA loses its stability to replicate (Morones et al., 2005). It also destabilizes the plasma membrane potential and results in the depletion of intracellular energy bond of ATP, thus resulting in cell death (Lok et al., 2006; Ramalingam et al., 2016). In the present set of experiments, incorporation of the ZnO-nanorods led to the growth promotion of *P. indica* which is supposed to act as media ingredients and carrier for the fast uptake of nutrients and gasses due to their small size, large surface area, and absorption capacity by the test fungus (Gleiter, 2000). Although, the various studies have been performed to understand the interaction between different nanoparticles and mycorrhizal fungi, but due to huge contradiction in the findings reported for each study. A lot of research has to be done to find out the exact role of different nanoparticles and their actual interaction with mycorrhizal fungi (Kotzybik et al., 2016).

## Conclusion

Zinc oxide nanorods have been successfully synthesized *via* mechanically assisted thermal decomposition method. In particular, ZnO-nanorods for the first time demonstrated the property of fungal symbiont productivity. Dry weight method has shown the maximum biomass of *P. indica* (about 60%) after interacting with optimized ZnO-nanorods (500 ppm) in comparison to control. Further, the interaction of *P. indica* with ZnO-nanorods significantly increases the number of fungal pellets, spore size, early sporulation, thick hyphae as confirmed by spore count method, scanning electron and confocal microscopic studies. Therefore, it is anticipated that the ZnO-nanorods on interaction with *P. indica* created a novel nanotool “nanoembedded fungus” which has the potential to significantly enhance the crop (*B. oleracea*) productivity as demonstrated in the present studies.

## Author Contributions

MK, RP, and AV: perceived and designed the experiments; US: conducted the experiments, MK, RP, and AV: analyzed the data; US: prepared the draft; RP, MK, and AV: proofread the final draft. All authors approved the final manuscript.

## Conflict of Interest Statement

The authors declare that the research was conducted in the absence of any commercial or financial relationships that could be construed as a potential conflict of interest.
